# Determination of Biogenic Amines in Wine from Chinese Markets Using Ion Chromatography–Tandem Mass Spectrometry

**DOI:** 10.3390/foods12112262

**Published:** 2023-06-04

**Authors:** Zuoyi Zhu, Xinyue Song, Yunzhu Jiang, Jiarong Yao, Zhen Li, Fen Dai, Qiang Wang

**Affiliations:** 1 Institute of Agro-Product Safety and Nutrition, Zhejiang Academy of Agricultural Science, Hangzhou 310021, China; zhuzuoyi2008@126.com (Z.Z.); jyunzhu@163.com (Y.J.); yao15356158878@163.com (J.Y.); lizhen0125@163.com (Z.L.); daif@mail.zaas.ac.cn (F.D.); 2 College of Chemical Engineering, Zhejiang University of Technology, Hangzhou 310014, China; m17857690676@163.com

**Keywords:** biogenic amines, wine, ion chromatography–tandem mass spectrometry, risk assessment

## Abstract

A method for the determination of nine biogenic amines (BAs) in wine was established using ion chromatography–tandem mass spectrometry (IC-MS/MS) without derivatization. BAs were separated by a cation exchange column (IonPac CG17, 50 mm × 4 mm, 7 µm) with a gradient aqueous formic acid elution. Good linearity was obtained for nine BAs with coefficients of determination (R^2^) > 0.9972 within the range of 0.01–50 mg/L. The limits of detection and quantification were within the ranges of 0.6–40 µg/L and 2.0–135 µg/L, respectively, with the exception of spermine (SPM). The recoveries were demonstrated within the range of 82.6–103.0%, with relative standard deviations (RSDs) of less than 4.2%. This simple method featuring excellent sensitivity and selectivity was suitable for the quantification of BAs in wines. The occurrence of BAs in 236 wine samples that are commercially available in China was determined. The BA levels in wines of different geographical origins varied significantly. The acute dietary exposure assessment of BAs was carried out by calculating the estimated short-term intake (ESTI) and comparing the acute reference dose (ARfD) specified by the European Food Safety Authority (EFSA). Results showed that the exposure to histamine (HIS) and tyramine (TYR) via the consumption of wines was much lower than the recommended ARfD level for healthy individuals. However, exposure could lead to symptoms in susceptible individuals. These results provided basic data regarding the occurrence and risk of BAs in wines for wine production, health guidance and consumer safety.

## 1. Introduction

Biogenic amines (BAs) are basic, non-volatile, nitrogenous organic compounds of low molecular weight [1]. According to the number of amine groups, BAs can be classed as monoamines, diamines or polyamines. BAs can also be either aliphatic (e.g., putrescine-PUT, cadaverine-CAD, spermine-SPM and spermidine-SPMD), aromatic (e.g., tyramine-TYR and 2-phenylethylamine-PHE) or heterocyclic (e.g., histamine-HIS and tryptamine-TRY) in terms of their chemical structure [2]. BAs are found in various kinds of foods and beverages, such as fishery, meat, vegetable and soybean products; cheese; wine; and beer [3,4,5]. Generally, BAs are formed from the decarboxylation of precursor amino acids via the actions of yeast, lactic acid bacteria or other contaminating microorganisms with amino acid decarboxylase activities [6].

High levels of BAs may lead to symptoms such as headache, hypertension, diarrhea, nausea, red rash and inflammation. HIS and TYR are considered the most harmful BAs, and they can act synergistically with PUT and CAD to aggravate poisoning symptoms, especially for susceptible individuals [7]. The consumption of ethanol, tobacco and monoamine-oxidase-inhibiting drugs increases an individual’s susceptibility [8]. In addition, the level of BAs is also regarded as a marker of sanitary conditions and microbial contamination degree in fermented foods [9]. The level of BAs in foods has become a concern for producers, consumers and enforcement authorities [10].

The presence of BAs in wine is common and inevitable [11]. There are two main factors influencing the content of BAs in wine: raw materials and the production process. Grapes of different varieties have different contents of free amino acids, resulting in different levels of BAs. During wine production, the level of BAs is usually low after alcoholic fermentation and increases dramatically after malolactic fermentation [12]. In addition, BAs can be accumulated during the wine aging and storage process, especially when a wine is exposed to microorganisms in the presence of free amino acids. Storage temperature, pH, oxygen and sulphur dioxide are important factors that influence the levels of BAs in wine [13,14,15].

It is widely recognized that BAs that are present in foods or beverages may trigger toxic symptoms. However, there is no shared legislation to limit the content of BAs in wine [16]. The recommended upper limit for HIS in wine is 2 mg/L in Germany; 3 mg/L in Holland; 5–6 mg/L in Belgium; 8 mg/L in France; and 10 mg/L in Switzerland and Australia [17]. Although wine is a widely traded product, most wine production involves a spontaneous fermentation process and is not regulated by specific standards [12]. All these are adverse for BA control in wine. Moreover, unlike other food products, the alcohol in wine can increase the potential toxicity of BAs, as ethanol contributes to BA transport and can inhibit the activity of amino oxidases [18]. These facts necessitate the limitation of BAs in wine. In view of the potential risk to human health, the standards aligned to controlling and monitoring BAs in wine are of great significance.

There have been a large number of studies carried out on BAs in wine from different countries, and these mostly aim to monitor the safety and quality of wine. However, there are only a few studies on BAs in wine with the purpose of determining the geographical origin or grape variety. Mitar et al. determined the contents of BAs in Croatian wine from two different wine-producing regions and found that the levels of BAs were associated with the geographical origin [19]. Ladente et al. found that the contents of BAs were related to grape varieties [20]. Marques et al. also investigated the relationship between BAs and grape variety in the red wine produced in three different Portuguese regions [21]. These studies suggested that BA composition was a feature of the geographical region. With respect to the risk assessment of BAs in wines, Esposito et al. evaluated the occurrence of BAs in wine that was commercialized in Italy and assessed the dietary exposure of BAs relative to consumers [22]. Han et al. profiled the occurrence of BAs in wine from Chinese markets and assessed the health risks [12]. Results showed that most wines exhibited a minor contribution to the recommended daily intake of BAs.

Numerous analytical methods have been reported for the determination of BAs, such as HPLC, CE, GC and IC. HPLC coupled to UV [18,23] or fluorescence detection [24,25] dominates among them. However, for HPLC analysis, chemical derivatization is usually performed using toxic reagents, such as dansyl chloride, benzoyl chloride or *o*-phthalaldehyde [26], due to the weak retention and the absence of a specific chromophore of BAs. IC is an alternative to the previously reported methods for BA analysis [27]. In contrast to HPLC approaches, IC offers simple sample pretreatment, improved retention and better chromatographic selectivity. IC with conductivity detection for the determination of BAs in food and beverage samples was presented by Palermo et al. [28]. In this case, no analyte derivatization is required, which is a great advantage for IC. However, conductivity detection is nonselective, interfering with BA quantification due to complex sample matrices. Furthermore, some BAs with low pKa values, such as TYR and TRY, have difficulty forming cations and cannot be detected by a conductivity detector. A similar IC approach for the determination of BAs in meat samples has been used by Favaro et al. [29] using integrated pulse amperometric detection, while the post-column addition of alkali is necessary before detection. IC coupled with MS detection offers solutions to the problems stated above and has been applied for the determination of BAs in processed meat products by Saccani et al. [30] and BAs in cheese by Scavnicar et al. [31]. According to our knowledge, there has been no method reported for the simultaneous determination of BAs in wine by IC-MS/MS.

In this work, a simple and rapid method for the determination of nine BAs in wine was developed. The separation of analytes was achieved using cation exchange chromatography, and the detection was performed by tandem mass spectrometry (MS/MS). This method not only simplified the sample’s pretreatment procedure without derivatization but also achieved good separation as well as high sensitivity and selectivity using MS/MS detection. Considering the great interest in the risk assessment of BAs in wine, the occurrence of BAs in 236 commercially available wine samples of different geographical origins in the Chinese market was investigated. This study may enrich the knowledge of the analytical methods of BAs and will be useful for the supervision of wine quality and safety, and it can establish the maximum allowable limit of BAs for wine in China.

## 2. Materials and Methods

### 2.1. Chemicals

Putrescine dihydrochloride (C_4_H_12_N_2_⋅2HCl, >98%), cadaverine dihydrochloride (C_5_H_14_N_2_⋅2HCl, >98%), histamine dihydrochloride (C_5_H_9_N_3_⋅2HCl, >99%), tyramine hydrochloride (C_8_H_11_NO⋅HCl, >99%), spermine tetrahydrochloride (C_10_H_26_N_4_⋅4HCl, >98%), spermidine trihydrochloride (C_7_H_19_N_3_⋅3HCl, >98%), tryptamine hydrochloride (C_10_H_12_N_2_⋅HCl, >98%), octopamine hydrochloride (C_8_H_11_NO_2_⋅HCl, >98%) and 2-phenylethylamine (C_8_H_11_N, >98%) were purchased from Shanghai Yuanye Bio-Chem Technology Co., Ltd., (Shanghai, China). Formic acid was of HPLC grade and purchased from Tedia Company (Fairfield, CA, USA). Ultrapure water (18.2 MΩ/cm) was prepared by a Millipore-Milli-Q system (Millipore, Shanghai, China). The other chemicals used in this study were of analytical grade.

### 2.2. Sample Collection and Preparation

A total of 236 commercial wine samples produced in different countries were collected and analyzed in this study. Of these wine samples, 213 were red wine samples and 23 were white wine samples according to the description on the label of the bottles. The origin of the red wine samples comprised China (*n* = 135), followed by France (*n* = 22), Australia (*n* = 19), Italy (*n* = 18) and other countries (Spain, Chile, Portugal, South Africa and America, *n* = 19). The origin of the white wine samples comprised Italy (n = 11), followed by the Czech Republic (*n* = 6) and China (*n* = 6). The majority of red wine was produced with grape cultivars comprising Cabernet Sauvignon, Marselan, Cabernet Gernischet, Merlot, Syrah and Cabernet Franc grape varieties. Regarding the white wine, most were produced with the Chardonnay grape variety.

Briefly, a 1 mL aliquot of each sample was transferred into a centrifuge tube, and 4 mL of 30 mM formic acid solution was added. Then, the diluted samples were filtered through a 0.22 µm pore size nylon filter before injection into the chromatographic system.

### 2.3. Preparation of Standard Solutions

The stock standard solutions of each BA were prepared at 10 mg/mL with a 30 mM formic acid solution and stored at –20 °C. The serial dilution of stock standard solutions was performed with a 30 mM formic acid solution to obtain mixed standard working solutions.

### 2.4. IC-MS/MS Analysis

Chromatographic separation was carried out on an LC-30AD HPLC system (SHIMADZU, Kyoto, Japan), and detection was performed with a tandem QTRAP5500 mass spectrometer (AB SCIEX, Framingham, MA, USA). The used column was a cation exchange guard column (IonPac CG17, 50 mm × 4 mm, 7 μm; Thermofisher, Sunnyvale, CA, USA) with a column temperature of 35 °C. The eluent consisted of 1.0 M aqueous formic acid (A) and ultrapure water (B). The linear gradient elution was as follows: 0.0 to 15.0 min, a linear gradient from 3% to 25% A; 15.0 to 16.0 min, a linear gradient from 25% to 100% A; 16.0 to 25.0 min, 100% A; 25.0 to 26.0 min, a linear gradient from 100% to 3% A; 26.0 to 30.0 min, 3% A. Analysis time was 25 min, and an additional 5 min was required for system equilibrium. The flow rate was 1.0 mL/min, and a flow divider valve was used before MS detection. The injection volume was 10 μL. MS analysis was carried out in the multiple-reaction monitoring (MRM) mode. Electrospray ionization was carried out in the positive ion mode. The MS parameters were as follows: ion spray voltage of 5500 V; gasification temperature at 500 °C; curtain gas at 40 psi; ion source gas at 50 psi and auxiliary gas at 60 psi. The conditions for MRM (declustering potential, entrance potential and collision energy) were optimized for each BA. The MRM transitions are listed in Table 1. Curve calibration and quantification were performed using the peak area of a single MRM transition.

### 2.5. Validation Procedure

The proposed method was validated by evaluating the validation parameters, including linearity, sensitivity, precision and accuracy. Linearity was assessed using calibration curves obtained after the injection of the mixed standard solutions of BAs with a wide concentration range. The coefficients of determination (R^2^) were calculated from the calibration curves. The method’s sensitivity was evaluated using the limits of detection (LODs, S/N = 3) and limits of quantification (LOQs, S/N = 10). Repeatability was tested by injecting the standard working solution six consecutive times. Reproducibility was evaluated by analyzing a spiked wine sample six consecutive times. The accuracy was determined via the triplicate analysis of spiked wine samples at different spiking levels.

### 2.6. Methodology of Risk Assessment

Acute exposure to BAs is more relevant than chronic exposure. Thus, the estimated short-term intake (ESTI) of HIS and TYR was calculated and compared with the acute reference dose (ARfD) specified by EFSA [10]. To evaluate the dietary exposure of BAs, data on the consumption of wine reported by EFSA [10] were used. The high consumption rates of red wine and white wine at the 95th percentile were 1000 and 1500 mL/meal, respectively. Two scenarios of exposure were considered: best case, where the median contents of HIS and TYR in the wines were used; worst case, where the contents of HIS and TYR at the 95th percentile were used.

The exposure of BAs was calculated as follows:

ESTI = C × Q

ESTI = Estimated short-term intake (mg/meal).

C = The 50th and 95th percentile contents of BAs in wines (mg/L).

Q = Individual wine consumption at the 95th percentile (L/meal).

### 2.7. Statistical Analysis

Microsoft Excel software was used to calculate the P5, P95, mean, median, max, standard deviation and RSD values. Principal component analysis (PCA) was performed using Origin 2018 software (Northampton, MA, USA).

## 3. Results

### 3.1. Optimization of IC-MS/MS Method

**Figure 1:**
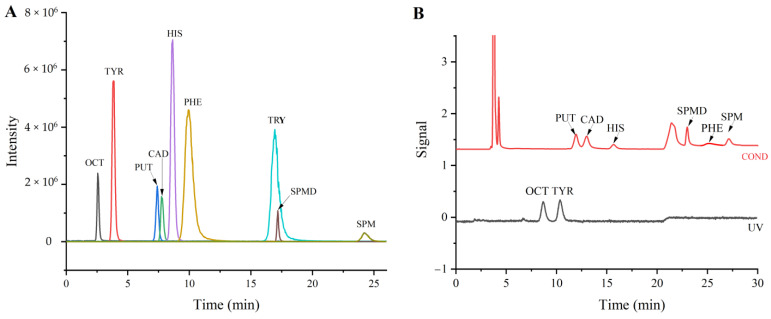
Typical chromatograms of a standard solution of nine BAs at a concentration of 1.0 mg/L (each analyte) by IC−MS/MS (**A**) and IC−conductivity/UV (**B**) detection.

During method development, the first thing to consider was the compatibility of IC separation and the MS detector. Usually, suppression is necessary for IC before using the MS detector if nonvolatile acid is used as the eluent. However, in this case, tyramine cannot be detected because it is removed by the suppressor [30]. In order to simultaneously determine common BAs, non-suppressed detection was used with a more volatile formic acid as the eluent in this study. The most used analytical column IonPac CS17 for the separation of BAs was initially tested. Because of the weaker elution power of formic acid compared with HCl, H_2_SO_4_ and methanesulfonic acid, some BAs were hardly eluted even with high concentrations of formic acid in the eluent. In order to obtain rapid analysis times, only the short CG17 guard column was used for the separation. The separation of nine BAs was achieved using a gradient elution of formic acid solution. The direct injection of the individual standard solution (1 mg/L) of each BA was performed to optimize mass conditions. Mass scans were carried out in the positive ion mode. After confirming the Q1 mass, two characteristic ion pairs were selected as qualitative and quantitative ions for each BA using a Q3 mass scan. Ionization conditions and collision energy were optimized for the best sensitivity (Table 1). A typical separation chromatogram for the mixed standard solution of nine BAs by MS/MS detection is shown in Figure 1A. All BAs were separated in 25 min. Even with the optimized elution program, there was still some peak overlapping for the partial co-elution of PUT and CAD and the complete co-elution of TRY and SPMD. However, the accurate quantification of the individual BA was obtained by using separate MRM fragments with the MS/MS detection system. Meanwhile, IC with suppressed conductivity and UV detection was used for comparisons. It was clearly shown that six BAs (PUT, CAD, HIS, PHE, SPMD and SPM) were detected using conductivity detection, and OCT and TYR were detected by UV detection, while TRY could not be detected by both conductivity and UV detection. The response signals of the BAs detected by conductivity and UV were much lower than those obtained by MS/MS detection (except SPM). The main reason for the low response signal of SPM by MS/MS was that the high concentration of formic acid (1 M) for the SPM elution suppressed ionization and reduced sensitivity.

### 3.2. Method Validation

The optimized method was validated in terms of linearity, sensitivity, precision and the accuracy of nine BAs. The analytical figures of merit for each BA are detailed in Table 2. Linear regression lines were obtained in a range from 0.01 to 50 mg/L for BAs, with coefficients of determination (R^2^) ranging from 0.9972 to 0.9998. The LODs and LOQs for eight BAs (except SPM) were within the ranges of 0.6–40 μg/L and 2.0–135 μg/L, respectively. Relatively high LODs (500 μg/L) and LOQs (1700 μg/L) were obtained for SPM due to the high concentration of the eluent. The repeatability of the developed method provided RSD values ranging from 0.9% to 2.4%, whereas the reproducibility of the method showed RSD values ranging from 0.6% to 3.4%. Good reproducibility results were probably due to the simple sample pretreatment with only one dilution step. The recovery percentage ranged from 82.6% to 101.9%, from 83.6% to 99.5% and from 84.9% to 103.0% in a selected wine sample using three different spiked levels, with RSD values lower than 4.2% (Table 3). All obtained recovery values fell within the range of −20% to +10%, indicating the good accuracy of the method.

### 3.3. Method Application

**Figure 2:**
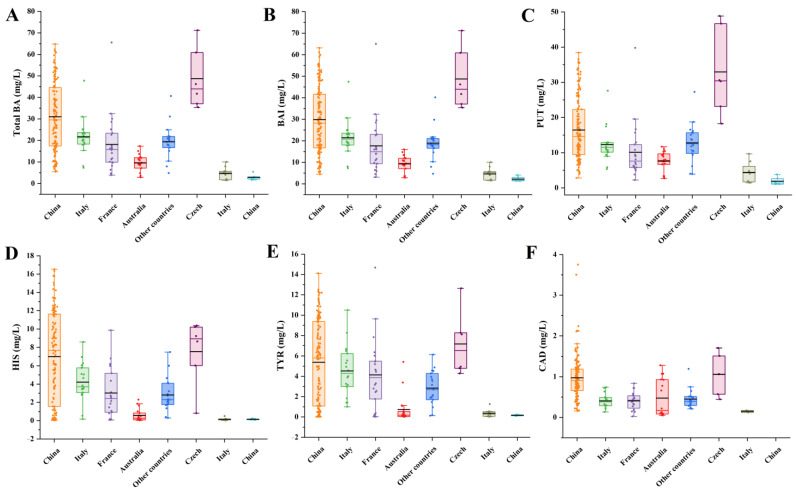
Box and whisker plot for total BA (**A**), BAI (**B**), PUT (**C**), HIS (**D**), TYR (**E**) and CAD (**F**) contents in wine samples of different geographical origins.

The validated IC-MS/MS method was applied to the quantification of BAs in 236 wine samples available in the Chinese consumer market. The total BA contents in 213 red wine samples were found to vary over a wide range from 2.86 to 65.51 mg/L. The obtained contents were consistent with a previous report in which the total BA levels in wines ranged from 1.2 to 52 mg/L [23]. As shown in Figure 2A, the red wines produced in China showed higher average BA levels at 31.02 mg/L than those from Italy (21.73 mg/L), France (18.18 mg/L), Australia (9.58 mg/L) and other countries (19.44 mg/L). The total BA content in 23 white wine samples ranged from 1.29 to 72.41 mg/L. Usually, red wine samples showed higher BA levels than those found in the white wine samples. However, extremely high BAs levels ranging from 35.41 to 71.19 mg/L were observed in six Czech white wine samples. Low BA levels with averages of 4.63 and 2.92 mg/L were found in Italian and Chinese white wine samples, respectively. The BA index (BAI), which is the sum of the contents of the given four BAs (PUT, CAD, HIS and TYR), was introduced to evaluate the quality of wines [32]. According to the results presented in Figure 2B, the BAI results corresponded to the total BA content, and a similar trend was observed in the analyzed wines of different geographical origins. This indicated that the major contributors to the total BAs in wine were PUT, CAD, HIS and TYR. For future wine-quality assessments, only the detection of PUT, CAD, HIS and TYR in wine samples may be enough, which greatly simplifies the analysis process of BAs.

In red wine, PUT, HIS, TYR and CAD were the most abundant BAs, with detection frequencies of 100%, 100%, 100% and 97%, respectively, for all samples. PUT was present within a content range between 2.23 and 39.77 mg/L, followed by HIS at 0.04−16.54 mg/L, TYR at 0.02−14.67 mg/L and CAD at LOQ−3.75 mg/L. The levels of PHE in 213 samples ranged from LOQ to 6.29 mg/L with a detection frequency of 99%, from LOQ to 0.21 mg/L for OCT with a detection frequency of 55%, from LOQ to 5.30 mg/L for SPMD with a detection frequency of 59% and from LOQ to 0.019 mg/L for TRY with a detection frequency of 1%. SPM was below the LOQ in any red wine sample. As shown in Figure 2C, PUT, which is associated with poor sanitary conditions, showed the highest content levels among nine BAs, and the trend of PUT levels in the red wine samples of different geographical origins was similar to that of the total BAs. PUT was found to fall within the range of 2.83−38.42 mg/L (average 16.44 mg/L) in the red wine samples produced in China, followed by Italy (5.36−27.60 mg/L, average 12.31 mg/L), France (2.23−39.77 mg/L, average 10.13 mg/L), Australia (2.71−11.71 mg/L, average 7.59 mg/L) and other countries (3.90−27.28 mg/L, average 12.79 mg/L). HIS is considered one of the most toxicologically relevant BAs in foods, and its levels in red wine produced in Australia (3.90−27.28 mg/L, average 12.79 mg/L) were lower than those from the other countries (Figure 2D). The distribution of TYR in the red wine samples of different geographical origins was similar to that of HIS (Figure 2E). Compared with PUT, HIS and TYR, the content levels of CAD were lower, and the average contents of CAD in red wine from different countries were not statistically significant from each other, with the exception of China (Figure 2F).

In white wine, PUT, HIS and TYR were the most abundant BAs, with a detection frequency of 100% for all 23 samples. PUT was present within a content range of 1.04 and 48.85 mg/L, followed by HIS 0.02−10.37 mg/L and TYR 0.02−12.64 mg/L. The levels of CAD in 23 samples ranged from LOQ to 1.71 mg/L with a detection frequency of 39% and from LOQ to 2.14 mg/L for PHE with a detection frequency of 96%. OCT and SPMD were present at low levels with a detection frequency of 17% and 43%, respectively. TRY and SPM were below LOQ in any white wine sample. As shown in Figure 2C, obvious differences were observed for PUT contents in the white wine samples of different geographical origins. PUT showed extremely high content levels (18.23−48.85 mg/L, average 32.97 mg/L) in Czech white wine samples and low levels (1.04−9.68 mg/L, average 3.29 mg/L) in Italian and Chinese white wine samples. The distributions of HIS, TYR and CAD in the white wine from three countries were similar to that of PUT. With the exception of Czech white wine samples, the contents of BAs in Italian and Chinese white wines were consistent with those reported in the literature [23].

**Figure 3:**
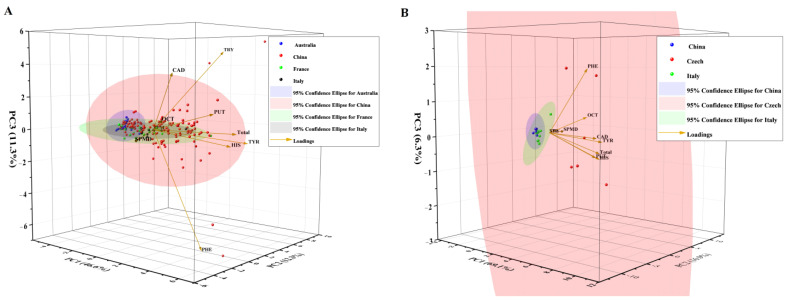
PCA biplot of red wine (**A**) and white wine (**B**) of different geographical origins.

PCA was employed to distinguish the wine samples of different geographical origins based on BA composition and content. For red wine, the first three principal components explained 70% of the total variability (Figure 3A). The first principal component PC1 was positively connected with PUT, OCT, Total BAs, TYR, HIS, SPMD and PHE. PC2 comprised a positive contribution of TRY, TYR, HIS and PHE and a negative contribution of PUT, CAD, OCT and SPMD. PC3 was positively related to TRY, CAD, PUT and OCT and negatively related to PHE. The red wine samples from China were distributed in multiple quadrants, showing a wide content range of BAs. Most red wine samples from Australia, France and Italy were placed together with some Chinese red wine samples due to their lower contents of BAs. The four wine groups were not well differentiated, indicating that the results of BAs in wines were not sufficient for geographical origin discrimination. For white wine, the first three principal components explained 92% of the total variance (Figure 3B). The first principal component, PC1, was positively connected to all individual amines. PC2 comprised a positive contribution of OCT, TYR and HIS and a negative contribution of PUT, CAD, PHE and SPMD. PC3 was positively related to PHE and OCT and negatively related to other BAs. The Czech wine samples were apparently placed in different confidence ellipses compared with Chinese and Italian white wines, which were placed together due to their low contents of BAs. The Czech white wine samples showed high contents of BAs, with two out of six samples related to PHE and one out of six samples linked with TYR, while three out of six samples exhibited high contents of HIS.

A comparison between our data and those reported by EFSA is shown in Table 4. The occurrence data (including mean, 5th percentile, median, 95th percentile and max values) for TYR, PUT, CAD, HIS and PHE in red and white wine samples obtained in this study were consistent with those reported by EFSA [10]. As reported, wine samples (including red, white and sparkling) reflected a mean value of 11.1 mg/L for the sum of BAs and a P95 value of 46.1 mg/L. In this study, the mean and P95 values for the total BAs in red wine were 25.96 mg/L and 53.72 mg/L, respectively, and the mean and P95 values for the total BAs in white wine (except Czech wines) were 4.12 mg/L and 8.61 mg/L, respectively.

### 3.4. Risk Assessment of BAs in Wine

So far, no legal limit has been established for the BA content in wine. Only some countries define the limit for HIS in wine, and it varies by country. The upper limit of HIS in wine is 2 mg/L in Germany; 3 mg/L in Holland; 5–6 mg/L in Belgium; 8 mg/L in France; and 10 mg/L in Switzerland and Australia [17]. Considering these limits, 20.7% of red wine samples in this study were found to have an HIS level higher than 10 mg/L and 67.1% of samples exhibited content higher than the strictest limit of 2 mg/L. Only white wine samples from Czech showed high HIS levels with an average of 7.55 mg/L. The HIS contents in white wine from Italy and China were all below 0.50 mg/L.

According to the EFSA risk assessment of BAs in fermented foods [10], the no-observed-adverse-effect level for HIS is 50 mg/meal for healthy individuals and zero for people with HIS intolerance. For TYR, it is 600 mg/meal for healthy individuals but only 50 mg for people taking third-generation monoamine oxidase inhibitor (MAOI) drugs and 6 mg for people taking classical MAOI drugs. The health risks of HIS and TYR that are involved in the consumption of wine samples investigated in this study were assessed. With respect to HIS, headaches, low blood pressure, heart palpitations, vomiting and diarrhea are the main side effects [7]. To evaluate the dietary exposure to HIS, the data on the consumption of wine reported by EFSA [10] were used. The high consumption rates of red wine and white wine at the 95th percentile are 1000 and 1500 mL/meal, respectively. For the occurrence data, P50 and P95 statistics for the contents of HIS in the wine of different geographical origins were used to estimate the risk. The combination of the consumption data multiplied with the HIS occurrence data was provided as the exposure value (ESTI) in mg/meal (Table 5). Overall, HIS and TYR exposure values varied for wines of different geographical origins. The upper HIS exposure values for red and white wine samples of 14.3 and 15.5 mg/meal were obtained in this study, and these are much lower than the HIS threshold of 50 mg/meal for healthy individuals and show a lower risk of experiencing HIS intoxication via the consumption of wine. However, the exposure values found in this study indicated that wine could lead to symptoms in people with HIS intolerance. As for TYR, the upper exposure values for red wine and white wine were 11.8 and 17.3 mg/day, respectively, far from the proposed toxic levels of 600 mg/meal for healthy individuals and 50 mg/meal for patients taking third-generation MAOI drugs. However, considering the EFSA’s limit of 6 mg/meal for patients taking classical MAOI drugs [10], the high risk comprising 34.7% of wine samples (mainly for red wine from China, Italy and France and white wine from Czech) investigated in this study cannot be overlooked.

## 4. Discussion

There is increasing interest with respect to analyzing BAs in foods and beverages. The HPLC separation technique is the most important instrumental method for a reliable analysis of BAs. A disadvantage of this method is the long and tedious sample pretreatment step, the necessity of pre- or post-column derivatization and the requirement of organic solvents. An alternative is IC using suppressed conductivity detection, which offers some advantages over the HPLC method for BA determination. These advances are mainly based on direct analysis without derivatization or preconcentration and a non-hazardous mobile phase. However, this method also has certain limitations and drawbacks. The complex matrix of food samples (most cations) interferes with the determination of BAs. In addition, some BAs with low pKa values have difficulty forming cations and cannot be detected by using a conductivity detector. Lately, there has been growing interest in the use of GC, HPLC and IC coupled to electrospray ionization (ESI) and mass spectrometry (MS). ESI ionizes the analytes directly prior to MS detection without derivatization treatments. Furthermore, the use of MS provides a significant improvement in the method’s sensitivity, selectivity and reliability of quantification. In this study, a reliable IC-ESI-MS/MS method was developed for the accurate determination of BAs present in wine. Compared with HPLC methods, the proposed method allowed the achievement of simple and eco-friendly procedures without using derivatization steps. Compared with IC-conductivity or UV detection, the analytical figures of merit obtained with the proposed method highlighted its competitiveness with respect to the number of determined BAs, sensitivity and selectivity. In this study, it can be observed in the validation results that the method showed a good linear range, repeatability, reproducibility and accuracy, which indicated that the proposed IC-MS/MS method was sufficiently suitable for the determination of BAs in wines.

The grape variety, winemaking process, specific strains and the composition of free amino acids are the main factors affecting the contents of BAs in wine and cause substantial differences in red and white wine [33]. Both maceration and malolactic fermentation are considered to increase the content of BAs in wine. These procedures are indispensable in red wine, but they are not common in the winemaking of white wine [34]. BAs were found at variable levels in wines depending on the grape variety; vintage; composition and levels of amino acids; processing techniques, such as malolactic fermentation; and storage [33]. The accumulation of BAs in wine is closely related to microbial ecology in wine fermentation, including yeasts and lactic acid bacteria exhibiting high decarboxylase activity [35]. Usually, red wine production includes maceration steps with grape skin. This process increases the contents of amino acids, which are the precursors of BAs. In addition, red wine production usually involves malolactic fermentation, which is not commonly applied to white wine production, explaining why the contents of BAs in red wine are higher than those in white wine.

Esposito et al. evaluated the dietary exposure to BAs via the consumption of wine commercialized in Italy, and results showed that no exposure values exceeded the levels of ARfD (50 mg/meal for HIS and 600 mg/meal for TYR) [22]. Han et al. also declared the low health risk of PUT, HIS and TYR in wine samples from the Chinese market [12]. Overall, most wine samples investigated in this study exhibited low exposure values compared with the ARfD of HIS and TYR. For healthy individuals, commercialized wine in China is considered safe for consumption. The obtained risk assessment results were consistent with those reported in the literature. However, as reported by Esposito et al., susceptible individuals may develop symptoms even at 4 mg per intake [22]. As reported, the co-presence of PUT, CAD and alcohol in wine could enhance the toxicity of HIS and TYR. Therefore, the risk of BAs in some groups exhibiting a high consumption of wine should not be ignored. In future studies, more extensive collections of toxicological data and extensive information campaigns are desirable. With the exception of effective methods for the control of BAs during winemaking, a regulatory system is urgently needed for different consumer groups with different susceptibility levels, such as creating a label with the values of BAs present in wine or specifying the absence of HIS and TYR in wine.

## 5. Conclusions

In this study, a simple, rapid and sensitive method for the determination of nine BAs in wines was established by IC-MS/MS without derivatization. The developed method showed excellent analytical performance in terms of linearity, sensitivity, precision and accuracy, indicating its suitability for the fast, accurate and environmentally friendly determination of BAs in wine. The occurrence of BAs in 236 wine samples available in the Chinese consumer market was investigated. Significant variations were found in the individual and total contents of BAs present in the wine of different geographical origins. The highest content of the individual BA was found to be that of PUT, followed by HIS, TYR and CAD in wines, proving that the introduction of BAI can be used to evaluate the quality of wine. The red wine samples investigated in this study showed higher BA levels than those found in the white wines. According to the risk assessment results of HIS and TYR, the exposure values were much lower than the levels of ARfD specified by EFSA. The wine available in the Chinese consumer market is considered safe for healthy individuals but may present risks in susceptible individuals with HIS intolerance or patients taking classical MAOI drugs. This study not only provided an efficient IC-MS/MS method for BA determination but also enriched information regarding the occurrence and risk of BAs in wine available in the Chinese market. All these are of great significance for wine production, health guidance and consumer safety.

## Figures and Tables

**Table 1:** IC-MS/MS conditions for the determination of BAs.

Compounds	Q1 Mass (Da)	Q3 Mass (Da)	Declustering Potential (V)	Entrance Potential (V)	Collision Energy (V)	Mobile Phase Composition
OCT	154.1	107.0 ^a^	23	9	26	Solvent A: 1.0 M formic acid; Solvent B: ultrapure water. The mobile phase was as follows: 0.0 to 15.0 min, a linear gradient from 3% to 25% A; 15.0 to 16.0 min, a linear gradient from 25% to 100% A; 16.0 to 25.0 min, 100% A; 25.0 to 26.0 min, a linear gradient from 100% to 3% A; 26.0 to 30.0 min, 3% A.
		91.0	31	10	35	
TYR	138.1	121.1 ^a^	30	10	13	
		77.1	24	10	36	
PUT	89.1	72.0 ^a^	31	8	13	
		30.0	30	9	30	
CAD	103.2	86.1 ^a^	30	9	12	
		69.0	40	10	20	
HIS	112.2	95.0 ^a^	53	10	18	
		68.0	60	10	28	
PHE	122.2	105.1 ^a^	26	9	15	
		77.0	37	9	16	
TRY	161.1	144.1 ^a^	25	7	14	
		117.1	30	8	32	
SPMD	146.2	112.1 ^a^	80	10	18	
		72.0	80	10	20	
SPM	203.2	129.1 ^a^	81	10	17	
		112.1	120	10	47	

^a^ Quantitative ion.

**Table 2:** Quantitative features of the developed IC-MS/MS method.

Analyte	Linearity Range (mg/L)	R^2^	LOD (μg/L)			LOQ (μg/L)			Repeatability, RSD%	Reproducibility, RSD%
			MS/MS	COND	UV	MS/MS	COND	UV		
OCT	0.01–5.0	0.9972	3.0	--	94	10	--	314	1.0	1.6
TYR	0.01–10	0.9986	0.6	--	100	2.0	--	340	0.9	1.4
PUT	0.1–10	0.9978	18	43	--	60	145	--	1.3	0.6
CAD	0.1–10	0.9989	20	51	--	68	170	--	1.1	1.3
HIS	0.01–10	0.9983	3.0	114	--	10	380	--	1.0	0.9
PHE	0.01–2.5	0.9998	1.5	220	--	5.0	735	--	1.8	2.1
TRY	0.01–5.0	0.9987	2.0	--	--	7.0	--	--	1.2	2.4
SPMD	0.2–10	0.9978	40	36	--	135	120	--	1.5	1.7
SPM	2.0–50	0.9986	500	98	--	1700	330	--	2.4	3.4

**Table 3:** Recoveries and precision of BAs spiked in the selected wine sample.

Analyte	Sample		Sample + 0.5 mg/L		Sample + 2.0 mg/L		Sample + 10 mg/L	
	Found (mg/L)	RSD%	Recovery%	RSD%	Recovery%	RSD%	Recovery%	RSD%
OCT	0.1	3.7	89.1	3.5	89.3	3.5	93.7	2.6
TYR	12.5	2.1	86.5	1.2	83.6	1.9	91.0	2.0
PUT	12.7	1.7	97.6	2.7	90.0	3.0	84.9	3.9
CAD	1.3	3.5	99.7	1.4	95.2	1.2	89.3	1.3
HIS	14.8	2.6	101.9	3.0	85.6	1.1	96.5	2.9
PHE	0.5	4.1	91.7	3.3	95.9	1.7	89.6	2.1
TRY	ND	--	88.9	4.0	96.0	1.6	96.2	2.4
SPMD	2.7	2.1	82.6	1.4	95.8	3.8	103.0	1.6
SPM	ND	--	--	--	99.5	4.2	95.9	3.4

**Table 4:** Occurrence data for BAs in wine (mg/L).

Reference	Category	BAs	n	ND	Mean	P5	Median	P95	Max
This study	Red wine	OCT	213	45%	0.055	0.030	0.048	0.11	0.21
		TYR	213	0%	4.54	0.027	3.83	11.38	14.67
		PUT	213	0%	14.33	4.26	11.95	31.59	39.77
		CAD	213	3%	0.79	0.14	0.73	1.62	3.75
		HIS	213	0%	5.40	0.091	4.36	13.36	16.54
		PHE	213	1%	0.33	0.026	0.18	1.04	6.29
		TRY	213	99%	0.016	0.014	0.016	0.018	0.019
		SPMD	213	41%	0.85	0.22	0.49	2.84	5.30
		SPM	213	100%	<2.5	<2.5	<2.5	<2.5	<2.5
		Total BAs	213	0%	25.96	6.63	21.95	53.72	65.51
		BAI	213	0%	25.03	5.85	21.20	52.40	65.02
	White wine	OCT	23	83%	0.047	0.039	0.048	0.054	0.054
		TYR	23	0%	2.08	0.025	0.26	8.25	12.64
		PUT	23	0%	11.04	1.06	4.06	45.07	48.85
		CAD	23	61%	0.76	0.13	0.57	1.63	1.71
		HIS	23	0%	2.09	0.066	0.18	10.11	10.37
		PHE	23	4%	0.40	0.038	0.17	2.01	2.14
		TRY	23	100%	<0.01	<0.01	<0.01	<0.01	<0.01
		SPMD	23	57%	0.98	0.25	0.79	2.12	2.19
		SPM	23	100%	<2.5	<2.5	<2.5	<2.5	<2.5
		Total BAs	23	0%	16.34	1.68	5.40	62.28	72.41
		BAI	23	0%	15.50	1.39	5.20	59.39	71.19
EFSA	Red wine	TYR	296	12%	2.7–2.9	<0.2	1.6–1.8	7.8–8.5	18.5
		PUT	120	5%	4.2–4.8	0.3–1	3.4–3.7	9.5–11.5	21.6
		CAD	126	26%	0.2–0.5	<0.1	0.1–0.2	0.6–1.6	5
		HIS	300	10%	3.6–3.7	<0.1	1.4–1.5	12.3–12.4	34.3
		PHE	24	100%	<2.4	<1.5	<1.5	<5	<5
	White wine	TYR	224	17%	1.1–1.2	<0.1	0.8	4.3–4.5	10
		PUT	100	3%	1.4–1.5	0.2	1.0	3.9–4.3	5.7–10
		CAD	100	30%	0.1–0.2	<0.1	<0.1	0.3–0.4	10
		HIS	225	22%	0.8–0.9	<0.1	0.3	2.6	55
		PHE	2	100%	<1.5	<1.5	<1.5	<1.5	<1.5

The table contains the number of samples (n), the percentage of non-detected (ND), the mean and several percentiles to describe the occurrence distribution (P5, P50 or median, P95 and max).

**Table 5:** Exposure to HIS and TYR with respect to the wine of different geographical origins.

Origin		HIS (mg/Meal)			TYR (mg/Meal)		
		Best Case	Worst Case	ARfD	Best Case	Worst Case	ARfD
Red wine	China	7.7	14.3	Healthy individuals: 50, People with HIS intolerance: 0	5.8	11.8	Healthy individuals: 600, People taking MAOI: 50, People taking classical MAOI: 6
	Italy	3.7	7.2		4.3	8.6	
	France	2.4	6.8		3.9	9.5	
	Australia	0.3	1.9		0.2	3.6	
	Other countries	2.3	6.2		2.7	5.0	
White wine	Czech	13.4	15.5		9.8	17.3	
	Italy	0.3	0.5		0.4	1.4	
	China	0.2	0.3		0.3	0.3	

## Data Availability

The data that support the findings of this study are available from the corresponding author upon reasonable request.
